# A finite element-based comparative dynamic biomechanical study of a novel oblique lateral locking plate system (OLLPS) versus traditional internal fixations for oblique lumbar interbody fusion (OLIF) under whole-body vibration

**DOI:** 10.3389/fbioe.2026.1826517

**Published:** 2026-07-17

**Authors:** Yinge Wang, Xiaoming Fu, Dan He, Shengju Zhu, Sha Tu, Dongyang Zou, Wei Wang

**Affiliations:** 1 Department of Spine Surgery, The 922nd Hospital of Joint Logistics Support Force, PLA, Hengyang, Hunan, China; 2 Department of Spine Surgery, Xiangnan Hospital, Hunan Normal University, Hengyang, Hunan, China; 3 Department of Pharmacy, The 921st Hospital of Joint Logistics Support Force, PLA, Changsha, Hunan, China; 4 Department of Nutrition, The 922nd Hospital of Joint Logistics Support Force, PLA, Hengyang, Hunan, China

**Keywords:** dynamic response, finite element analysis, novel fixation, OLIF, whole-body vibration

## Abstract

**Objective:**

To evaluate the biomechanical performance of the novel OLLPS in OLIF surgery, including its dynamic stability and application safety, thereby providing evidence-based support for optimizing OLIF combined internal fixation strategies.

**Methods:**

Five surgical finite element analysis models with OLLPS or traditional internal fixation were established based on a validated L1-S1 intact model. A 400N follower load was applied along the lumbar curvature to simulate partial body weight; simultaneously, a 5Hz, ±40N sinusoidal vertical load was added to L1 vertebral upper surface to simulate whole-body vibration (WBV) during driving on regular paved roads, with a loading duration of 2 s. Post-loading, peak dynamic response parameters of surgical and adjacent segment structures were extracted, and their maximum and minimum values and vibration amplitudes were recorded.

**Results:**

Bilateral pedicle screw fixation (BPS) provides the greatest reduction in surgical segment endplate stress (59.41%–60.35% reduction in L5 superior endplate) and cage stress (a maximum reduction of 68.19%), but induces the highest increases in adjacent segment (especially L5/S1) intervertebral disc stress (IDS) and facet joint stress (FJS). OLLPS effectively reduces surgical segment stress; despite its relatively high implant stress, it caused less adjacent segment interference than BPS. 2-screw and 4-screw lateral plate fixations had weaker stress-modulating effects than BPS and OLLPS. Additionally, the IDS and FJS at L3/4 generally exceeded those at L5/S1, and the IDS and FJS at L5/S1 were more strongly influenced by the fixation scheme.

**Conclusion:**

In OLIF, different supplemental fixations induce differing dynamic biomechanical responses at surgical and adjacent levels. Compared with traditional internal fixations, OLLPS based on locking structure and reverse pedicle screw trajectory design provides balanced biomechanical compatibility in stability and adjacent-segment disturbance. The fixation scheme with appropriate stiffness should be prioritized based on patients’ adjacent-segment degeneration status to reduce the related risks.

## 1 Introduction

Degenerative lumbar diseases (e.g., disc herniation, stenosis, spondylolisthesis) are common in spinal surgery. Lumbar interbody fusion is a classic treatment, and minimally invasive oblique lumbar interbody fusion (OLIF) has emerged as a research focus due to its unique advantages (Gutierrez et al., 2025). OLIF achieves indirect neural decompression by restoring intervertebral height and lumbar curvature, while reconstructing spinal alignment (Yu, 2024). Via a retroperitoneal approach between the abdominal aorta and the left psoas major, it avoids anterior vascular injury risks and reduces posterior complications (e.g., myogenic low back pain), with shorter operative time, less blood loss, and faster recovery (Chang et al., 2024; Chen et al., 2024; Dada et al., 2025; Gutierrez et al., 2025; Tsuchiya et al., 2025). However, the efficacy of OLIF depends on the stability of large intervertebral cages; cage subsidence, a major complication, is more common in patients with osteoporosis, endplate injury, or inappropriate fixation selection, and may lead to intervertebral space collapse, spinal canal restenosis, and recurrent nerve compression (Kotheeranurak et al., 2023; Shen et al., 2024; Yang et al., 2024; Chang et al., 2025). OLIF combined with bilateral pedicle screw-rod fixation (BPS) can reduce this risk but requires intraoperative patient repositioning and additional incisions, which prolongs operative time, increases anesthesia-related and paraspinal muscle injury risks, raises medical costs, and may induce adjacent segment disease due to excessive segmental rigidity (Du et al., 2021; Cai et al., 2023; Kang et al., 2023; Chou et al., 2025; Dai et al., 2025).

**FIGURE 1:**
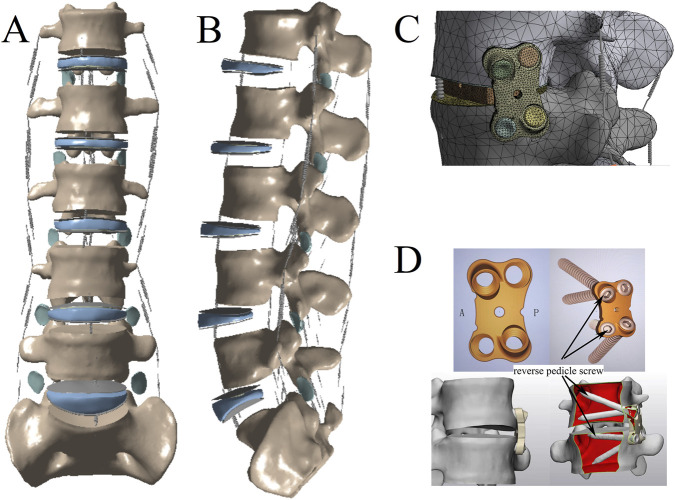
**(A)** and **(B)** Anatomical structure separation views of L1-S1 finite element model (anterior and lateral projections). **(C)** Schematic diagram of mesh generation details. **(D)** Schematic diagram of OLLPS and reverse pedicle screw trajectory.

To address these limitations, lateral fixation strategies for OLIF have been explored (DenHaese et al., 2021; Song et al., 2021; Zhang et al., 2023; Dai et al., 2025; Li S. et al., 2025). Inspired by Sardhara et al.‘s Reverse Pedicle Screw Fixation (RPSF) (Sardhara et al., 2019), our team integrated Biological Osteosynthesis (BO) principles to develop the innovative Oblique Lateral Locking Plate System (OLLPS; Patent No.: ZL 202022949889.1, Figure 1D). The structurally optimized OLLPS locking plate, along with four multi-axial screws (including two reverse pedicle screw trajectories), forms a three-dimensional scaffold that provides angular stability and stress dispersion. Our static biomechanical study confirmed that OLLPS matches BPS in range-of-motion (ROM) restriction and outperforms other traditional lateral fixations (Wang et al., 2022).

With the increasing demand for rapid postoperative recovery, driving or riding in vehicles is common and inevitable for patients in the early postoperative period after lumbar fusion, exposing them to whole-body vibration (WBV), a well-established risk factor for spinal disorders (Zhang et al., 2024). Compared with static loading, cyclic WBV increases vertebral stress and intradiscal pressure, accelerates spinal degeneration, and may induce fatigue damage to spinal structures and implants (Burström et al., 2015). Intervertebral disc degeneration alters lumbar vibrational characteristics, further amplifying the adverse effects of WBV (Guo and Fan, 2017). Finite element simulation is critical for investigating WBV-induced biomechanical responses. WBV’s impact on intact lumbar spines was quantified via finite element analysis showing that under 40 N equivalent amplitude vibrational loading, lumbar axial displacement and intradiscal pressure increased by 314.5% and 242.4% versus static loading, respectively (Guo and Fan, 2018). Previous finite element studies confirm distinct biomechanical behaviors of different lumbar fusion procedures under WBV (Fan and Guo, 2019; Fan et al., 2021; Xie et al., 2025). Posterior lumbar interbody fusion (PLIF) and transforaminal lumbar interbody fusion (TLIF) exhibit higher endplate stress at the operative segment, elevating cage subsidence risk. OLIF promotes bone healing via anterior load shifting but increases facet joint stress and adjacent segment degeneration risk. Anterior lumbar interbody fusion (ALIF) provides excessive rigid stability with significant stress shielding, potentially aggravating bone brittleness in osteoporotic patients long-term. In the early postoperative period, solid bony fusion has not yet been achieved, and the operated segment remains mechanically unstable and highly susceptible to WBV stimulation (Fan and Guo, 2021). Appropriate selection of internal fixation implants is therefore critical to maintaining postoperative spinal stability and reducing the incidence of complications. Nevertheless, most existing studies have mainly focused on the dynamic biomechanical performance of conventional fusion approaches and traditional internal fixation constructs under WBV loading. Few investigations have addressed the dynamic stability of OLIF combined with novel internal fixation systems such as OLLPS. This study employs finite element analysis to evaluate the dynamic biomechanical performance of OLIF combined with OLLPS versus traditional internal fixations under WBV conditions in the early postoperative period, aiming to assess the dynamic stability and safety of OLLPS, thereby providing evidence for optimizing clinical fixation strategies and promoting minimally invasive lumbar fusion techniques.

## 2 Materials and methods

### 2.1 Finite element models and implants

Ligaments and facet joints are critical for lumbar stability and vibration buffering (Widmer et al., 2020). To improve computational accuracy, this study used a validated full-element three-dimensional lumbar finite element model established by our research group. The model included vertebral bodies (cortical bone, cancellous bone, posterior osseous structures), endplates, intervertebral discs (nucleus pulposus, annulus fibrosus), facet joints, and major ligaments (Figures 1A,B). Ligaments were defined as tension-only spring elements. All structures except ligaments were meshed with tetrahedral and hexahedral elements (Figure 1C). Based on the well-established finite element modeling methods in previous studies (Chayer et al., 2025; Li X. et al., 2025), elastic material properties were assigned to bone tissue, intervertebral discs and implants. Following the same modelling assumptions, all structures were defined as homogeneous, continuous and isotropic, with mechanical behaviours characterised by Young’s modulus and Poisson’s ratio. Detailed material parameters are summarized in Table 1 (Zhang et al., 2018; Guo and Wang, 2020; Kumaran et al., 2021; Shen et al., 2021b). Mesh sensitivity analysis was performed, and the global element size was set to 1.5 mm. The intact model contained 511,826 elements and 276,996 nodes. This model has been verified by range-of-motion and stress tests and reliably reflects *in vivo* lumbar biomechanics.

**TABLE 1:** Material properties of the finite element model components.

Structure	Young’s modulus (MPa)	Poisson’ ratio	Cross- sectional area (mm^2^)	Density (kg/mm^3^)
Bone				
Cortical bone	12,000	0.3	​	1.7e-6
Cancellous bone	100	0.2	​	1.1e-6
Endplate	500	0.25	​	1.2e-6
Intervertebral disc				
Nucleus pulposus	1	0.49	​	1.02e-6
Annulus fibrosus	4.2	0.45	​	1.05e-6
Ligaments				
Anterior longitudinal	20	0.3	63.7	1.0e-6
Posterior longitudinal	20	0.3	20	1.0e-6
Ligamentum flavum	19.5	0.3	40	1.0e-6
Interspinous	11.6	0.3	40	1.0e-6
Supraspinous	15	0.3	30	1.0e-6
Intertransverse	58.7	0.3	3.6	1.0e-6
Capsular	32.9	0.3	60	1.0e-6
Implants				
Pedicle screws and rod (Ti-6A1-4V)	110,000	0.3	​	4.5e-6
Lateral plate and screws (Ti-6A1-4V)	110,000	0.3	​	4.5e-6
Cage(PEEK)	3500	0.3	​	1.32e-6

**FIGURE 2:**
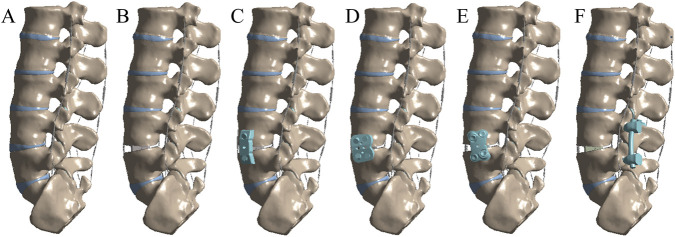
Finite element models of OLIF with different internal fixations. **(A)** Intact, **(B)** SA **(C)** LP-2, **(D)** LP-4, **(E)** OLLPS, **(F)** BPS.

Based on our previous method (Wang et al., 2022), five surgical models were constructed at L4/5 (Figure 2). A stand-alone OLIF (SA) model was constructed using the CLYDESDALE cage (Medtronic Sofamor Danek USA Inc., Memphis, Tennessee, USA), featuring an anterior convexity of 6°, a length of 50 mm, a width of 18 mm, an anterior height of 12 mm, and composed of polyetheretherketone (PEEK). All internal fixation models were constructed based on the SA model. The OLIF with 2-screw lateral plate (LP-2) model utilized the Pivox Oblique Lateral Spinal System (Medtronic Sofamor Danek USA Inc.), with a plate measuring 34.6 mm in length, 12 mm in width, and 5.4 mm in thickness, accompanied by screws with a length of 45 mm and an outer diameter of 5.5 mm. The OLIF with 4-screw lateral plate (LP-4) model was built using the LITe plate system (Stryker USA Inc., Kalamazoo, Michigan, USA), featuring a plate with a length of 28 mm, a width of 21 mm, and a thickness of 4.5 mm, along with screws measuring 45 mm in length and 5.5 mm in outer diameter. The OLIF with OLLPS model incorporated a plate with a length of 32 mm, a width of 22 mm, and a thickness of 5 mm, plus screws with a length of 45 mm and an outer diameter of 6.0 mm. The OLIF with BPS model was constructed based on the CDHSEXTANT II (Medtronic Sofamor Danek USA Inc.), with pedicle screws having an outer diameter of 6.5 mm and a length of 45 mm, and a connecting rod with a diameter of 5.5 mm. All implants were titanium alloy (Ti6Al4V); material properties are shown in Table 1.

### 2.2 Boundary and loading conditions

All finite element simulations were performed using ANSYS Workbench (Ansys Inc., Canonsburg, PA, USA) on high-performance workstations equipped with dual Intel Xeon processors. Non-separate contact was adopted between the intervertebral disc/cage and the vertebral endplates. Given the anti-migration tooth structure on the cage surface, the friction coefficient at the cage-endplate interface was set to 0.8 to simulate the immediate postoperative condition before bone fusion. A friction coefficient of 0.2 was applied to the facet joint surfaces and screw-bone interfaces. The OLLPS screw-plate interfaces were modeled with non-sliding contact to represent the locking mechanism, in contrast to the friction coefficient of 0.2 used in traditional screw-plate interfaces (Areias et al., 2020). All nodes on the S1 inferior surface were fully constrained in all translational and rotational degrees of freedom to simulate pelvic fixation under the upright physiological posture.

Given the inherent limitations of finite element numerical calculation, prolonged computation over weeks or months is impractical. Therefore, short-term cyclic WBV loading was used in this study to extrapolate the biomechanical performance of the lumbar spine under long-term repetitive loading. This simplified cyclic loading protocol is a well-recognized and standard modeling strategy commonly adopted in spinal biomechanics research. Following the loading protocols reported in previous studies, a 400 N follower load was applied along the lumbar curvature to simulate partial body weight. Simultaneously, a sinusoidal vertical load with a frequency of 5 Hz and an amplitude of ±40 N was applied to the L1 superior surface to simulate WBV exposure during driving on regular paved roads (Fan and Guo, 2021; Fan et al., 2021; Xie et al., 2025). The loading duration was set to 2 s, with data sampled at an interval of 0.05 s. After loading, peak dynamic response parameters were extracted: stress distributions at the superior and inferior endplates of the surgical segment, cage stress, internal fixation stress, and intervertebral disc stress (IDS) and facet joint stress (FJS) at adjacent segments (L3/4 and L5/S1). The maximum value (Max), minimum value (Min), and vibration amplitude (VA, Max-Min) of each parameter were recorded. The biomechanical performance of each internal fixation system under the dynamic WBV condition was evaluated.

## 3 Results

### 3.1 Endplate stress at surgical segment (L4/5)

**FIGURE 3:**
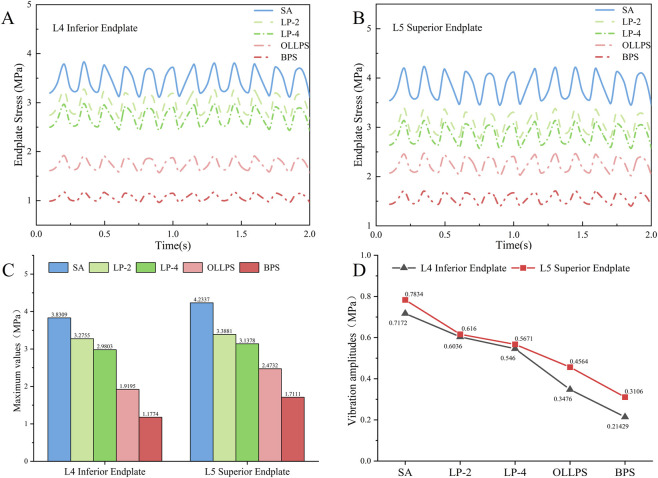
Comparative analysis of endplate stress dynamics under different internal fixation methods, showing the dynamic response of the **(A)** L4 inferior and **(B)** L5 superior endplates, along with their corresponding **(C)** maximum values and **(D)** vibration amplitudes.

**FIGURE 4:**
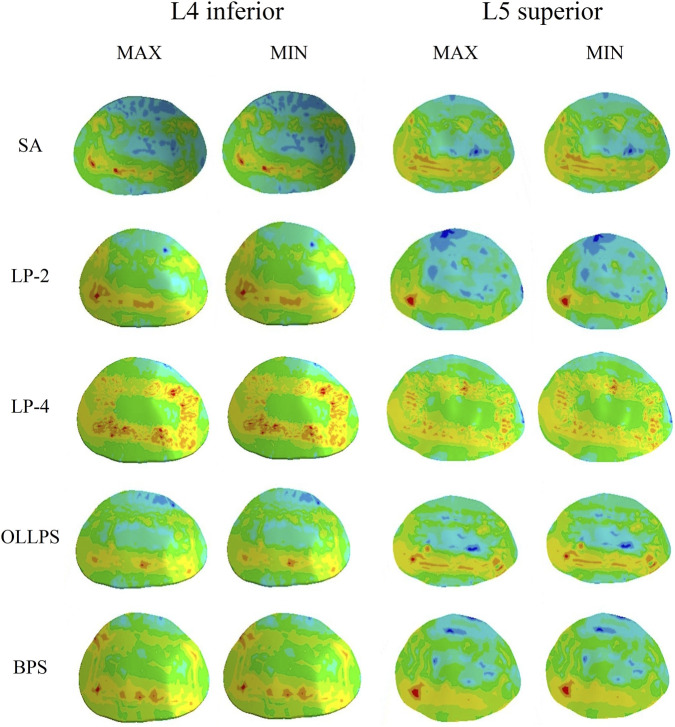
Stress distribution on the L4 inferior and L5 superior endplates at the peak (red region) of the dynamic response under different fixation methods.

The endplate stress at the L4/5 segment exhibited high sensitivity to vibrational loading across different internal fixation methods, with stress distribution patterns closely associated with the risk of cage subsidence (Kim et al., 2022). Figures 3, 4 illustrate the time-domain dynamic stress responses of the L4 inferior and L5 superior endplates under WBV. The response curves exhibited clear cyclic variation over time. Within the observed time window, Max, Min, and VA are the key evaluation metrics. After internal fixation, both the L4 inferior and L5 superior endplates exhibited consistent trends, with decreases in overall stress levels and vibration amplitudes. In the SA model, Max, Min, and VA at the L5 superior endplate were 4.2337 MPa, 3.4503 MPa, and 0.7834 MPa, respectively. Compared with the SA model, the OLLPS system reduced these values by 41.58%, 41.55%, and 41.74%, respectively. The BPS system demonstrated the most pronounced vibration-damping effect, achieving reductions of 59.58%, 59.41%, and 60.35% in Max, Min, and VA, respectively. As shown in Figure 4, under WBV loading, high stress concentration areas (indicated in red) were predominantly localized at the cage-endplate interface.

### 3.2 Implant stress

**FIGURE 5:**
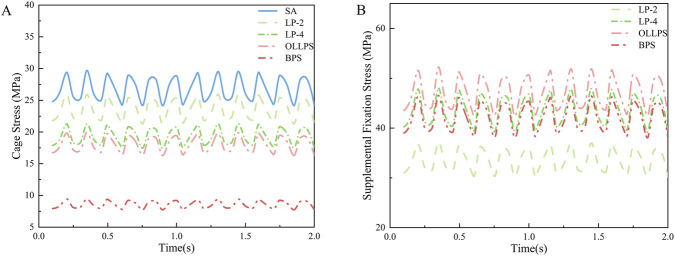
Comparative analysis of dynamic stress in the cage and supplemental fixations, shown in **(A)** and **(B)**, respectively.

Implant stress is clinically relevant due to its association with mechanical failure, such as cage damage or internal fixation fracture. Reported material properties indicate a tensile yield strength of 110 MPa for PEEK cages, while Ti-6Al-4V implants exhibit a fatigue strength of 500 MPa and a yield strength between 795 and 827 MPa (Wang and Guo, 2021; Guo et al., 2025). In the present study, the SA model exhibited the most pronounced dynamic cage stress response, with Max, Min, and VA of 29.6890 MPa, 24.1490 MPa, and 5.5400 MPa, respectively. Both LP-2 and LP-4 reduced cage stress to some extent, whereas OLLPS and BPS demonstrated more substantial stress-shielding effects (Table 2; Figure 5). Relative to the SA model, the OLLPS system reduced Max, Min, and VA of cage stress by 32.75%, 32.56%, and 33.59%, respectively. The BPS system achieved even greater reductions of 68.15%, 67.99%, and 69.03%. With respect to internal fixation stress, the OLLPS system sustained the highest stress, with Max, Min, and VA of 52.2240 MPa, 42.3700 MPa, and 9.8540 MPa, respectively, and the peak stress was localized on the plate. The LP-2 and LP-4 models exhibited lower dynamic stress levels than OLLPS and BPS, with stress concentrations occurring at the screw-plate interface. In the BPS model, the corresponding internal fixation stress values were 46.5260 MPa, 38.0150 MPa, and 8.5110 MPa, with the peak stress located on the rods.

**TABLE 2:** The dynamic responses of the implants under WBV.

Dynamic response	SA			LP-2			LP-4			OLLPS			BPS		
	Max	Min	VA	Max	Min	VA	Max	Min	VA	Max	Min	VA	Max	Min	VA
Cage stress (MPa)	29.6890	24.1490	5.5400	25.9700	21.2620	4.7080	21.3020	17.4310	3.8710	19.9660	16.2870	3.6790	9.4453	7.7296	1.7157
Supplemental fixation stress (MPa)	-	-	-	37.2790	30.2040	7.0750	48.0090	39.2220	8.7870	52.2240	42.3700	9.8540	46.5260	38.0150	8.5110

### 3.3 Adjacent segment intervertebral disc stress

**FIGURE 6:**
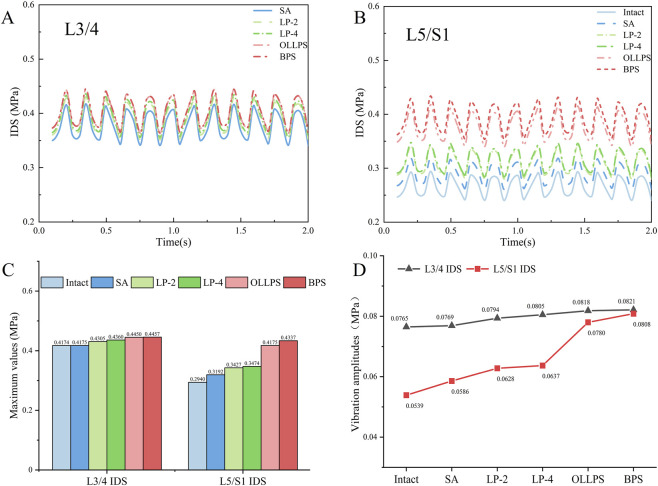
Dynamic response of adjacent intervertebral disc stress under different fixation methods. **(A)** Stress-time curve at L3/4, **(B)** Stress-time curve at L5/S1, **(C)** Maximum stress, **(D)** Vibration amplitude.

Previous studies have consistently reported that lumbar fusion elevates mechanical stress in adjacent segment discs, thereby accelerating degenerative processes (de Andrada Pereira et al., 2022). As shown in Figure 6, compared with the adjacent discs (L3/4 and L5/S1) in the intact model, all surgical models exhibited increased disc stress, with the BPS model showing the most pronounced elevation. Building on findings from our earlier static biomechanical analysis, the current dynamic results further support that greater stabilization of the surgical segment correlates with a more marked increase in stress on adjacent discs. At the L3/4 level, differences in disc stress among the models were modest, with response values remaining relatively consistent across groups, though uniformly higher than those at the L5/S1 level under equivalent fixation conditions. In contrast, postoperative stress values at the L5/S1 disc varied more substantially across models, indicating that the choice of surgical and fixation techniques differentially influences mechanical loading at this level. In the BPS model, Max, Min, and VA of L5/S1 disc stress increased by 47.52%, 46.94%, and 46.99%, respectively, relative to the intact model. Corresponding increases in the OLLPS model were 42.00%, 41.37%, and 41.43%.

### 3.4 Adjacent segment facet joint stress

**FIGURE 7:**
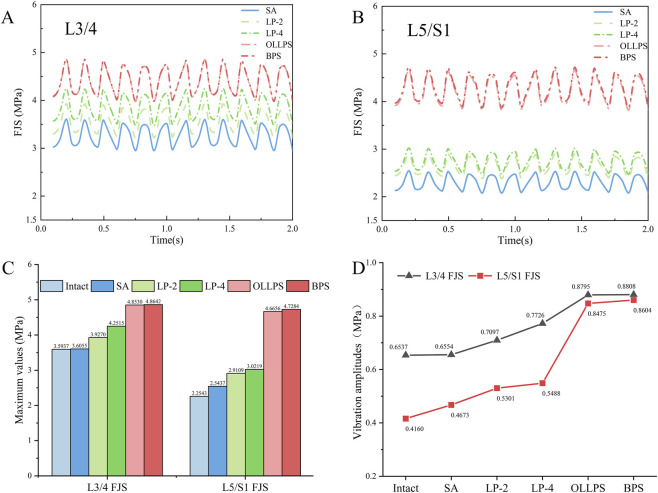
Dynamic response of adjacent facet joint stress under different fixation methods **(A)** Stress-time curve at L3/4, **(B)** Stress-time curve at L5/S1, **(C)** Maximum stress, **(D)** Vibration amplitude.

The lumbar facet joints play an important role in maintaining spinal stability and contribute to multi-directional coupled motion. In the present study, dynamic stress responses of the facet joints in the model are presented in Figure 7. At the L3/4 level of the intact model, Max, Min, and VA were 3.5937 MPa, 2.9400 MPa, and 0.6537 MPa, respectively; at the L5/S1 level, the corresponding values were 2.2543 MPa, 1.8383 MPa, and 0.4160 MPa. After surgery, facet joint stress at both adjacent segments rose in all instrumented models. Such stress increased most substantially in the OLLPS and BPS constructs, especially at the L5/S1 level, when compared with the SA, LP-2 and LP-4 models. Relative to the intact condition, the BPS model increased Max, Min, and VA at L5/S1 by 109.75%, 110.41%, and 106.82%, respectively. The OLLPS system induced comparatively lower stress elevations than BPS.

## 4 Discussion

Whole-body vibration (WBV) represents a form of dynamic, cyclic mechanical loading that can induce micromotion, stress concentration, and potential fatigue failure at the bone-implant interface, thereby influencing the initiation and progression of spinal fusion. During the early postoperative period after OLIF, prior to the attainment of solid bony fusion, the dynamic response provided by different internal fixation systems under WBV, which include their capacity to resist cage subsidence and their potential impact on adjacent segments, serves as a critical metric for evaluating overall construct performance. However, this area remains insufficiently investigated in the existing literature. The present study, therefore, applied finite element analysis to examine these biomechanical characteristics systematically.

### 4.1 Distinct biomechanical insights from whole-body vibration versus static loading

WBV is fundamentally distinct from static multi-directional testing in terms of mechanical input and tissue response characteristics. Static simulation generally applies eccentric pure moment to evaluate directional stability and load distribution under quasi-physiological postures (Shen et al., 2021a; Xie et al., 2025). Such tests can only capture the instantaneous mechanical response under a fixed posture. In contrast, WBV applies sinusoidal vertical excitation at a frequency close to the first resonance mode of the lumbar spine (approximately 5 Hz), which further amplifies the dynamic response through inertial and elastic effects and represents a continuous mechanical stimulation (Guo and Fan, 2018). Existing studies have demonstrated that vibration loading with the same amplitude induces far greater peak-to-valley variations in disc bulging, intradiscal pressure and annulus fibrosus stress than static loading. In the intact spine, such amplification effect exceeds 200% (Guo and Fan, 2018) and varies with the stiffness of internal fixation constructs (Fan and Guo, 2021; Zhu et al., 2022). This discrepancy arises from the fact that WBV excites the frequency-dependent mechanical impedance of the spine and triggers a cyclic energy absorption process that cannot be replicated by static testing (Guo and Fan, 2018; Fan and Guo, 2019).

In the internal fixation-assisted fusion model, WBV analysis reveals time-domain phenomena particularly relevant to clinical scenarios such as vehicle vibration exposure. The relative performance ranking of different fixation strategies under WBV may differ from that predicted by static tests. The U-shaped titanium alloy structure of the Coflex-F device allows interspinous micromotion and dissipates vibration energy through elastic deformation and interface friction. Compared with BPS, it reduces the amplitude of facet joint stress at adjacent segments by 22%–35% and the amplitude of nucleus pulposus pressure by 36%–46% (Zhu et al., 2022). Such damping superiority is insignificant in static tests and can only be activated under cyclic dynamic loading. The titanium alloy cage used in ALIF possesses a much larger modulus (113,000 MPa), leading to severe modulus mismatch with bone tissue. The strain energy in the bone graft region under vibration is only 4.5 kJ/m^3^. Long-term stress shielding may accelerate the progression of osteoporosis in the fusion region, and static testing fails to capture such time-domain energy distribution imbalance as well as its long-term effects on osseointegration (Xie et al., 2025). The overall spinal vibration sensitivity decreases after fusion surgery, whereas local loading at adjacent segments may increase. A previous study demonstrated that BPS increases facet joint stress at adjacent segments by 4%–10% (Shen et al., 2021a). WBV is capable of capturing cumulative tissue responses and resonance-driven stress concentration that cannot be obtained by conventional static or single-cycle evaluation, providing supplementary insights into the durability of internal fixation constructs under repeated physiological exposure.

### 4.2 Dynamic response mechanisms at the surgical segment and clinical correlations

In the surgical segment, the dynamic characteristics of endplate/implant stress are core indicators for evaluating the risks of cage subsidence and internal fixation failure after OLIF. As a periodic dynamic load, the effects of WBV on endplate and implant stress are not a simple superposition of static loads. Cage subsidence is one of the primary causes of revision surgery following stand-alone OLIF (CreveCoeur et al., 2023; Chou et al., 2025). The higher stress at the interface between the cage and endplate corresponds to a greater risk of endplate damage, fracture, and intervertebral cage subsidence (Kim et al., 2013; Kim et al., 2022; Chou et al., 2025). In our study, all surgical models exhibited higher endplate stress peaks than the intact model under dynamic conditions. Stress concentrations were consistently observed at the cage-endplate interface, with the L5 superior endplate experiencing higher stress levels than the L4 inferior endplate across all instrumented models. This biomechanical finding, which is consistent with the studies by Xie et al. and Tao et al. (Tao et al., 2024; Xie et al., 2025), consequently suggests a higher risk of stress-induced damage to the L5 superior endplate. The predisposition of the superior endplate to failure can be explained by anatomical evidence: the superior endplate of a vertebra is consistently thinner and structurally weaker than its inferior counterpart, rendering it more vulnerable to deformation and damage (Zhao et al., 2009; Wang et al., 2011). This mechanism is strongly corroborated by clinical imaging studies. Wu et al. reported that the majority of cage subsidence cases (37 segments, 34.6%) occurred at the superior endplate (Wu et al., 2023). Similarly, Hu et al. found a subsidence incidence of 35.3% at the superior endplate versus only 5.0% at the inferior endplate (Hu et al., 2023). Furthermore, clinical evidence confirms that most subsidence occurs within the first 6 months postoperatively, with a predilection for the endplate below the cage (Le et al., 2012; Marchi et al., 2013; Hu et al., 2023; Hu et al., 2025). This critical period coincides with the pre-fusion phase, during which the endplate exhibits heightened vulnerability to dynamic loads. Our finite element stress nephograms further revealed that peak dynamic stresses concentrated at the dorsal cage-endplate interface, suggesting this region as a potential initiation site for structural compromise. This stress distribution pattern, coupled with the inherent anatomical vulnerability of the endplate, underscores the importance of careful surgical handling of the dorsal endplate region. It also suggests a potential role for targeted endplate reinforcement strategies—such as the sub-endplate support screws incorporated in the OLLPS design—in mitigating subsidence risk (Wang et al., 2022; Han et al., 2025; Nong et al., 2025).

Different internal fixation systems exhibit varying effects on regulating the stress of endplates and cages, and this difference essentially stems from the distinctions in mechanical transmission path design and stiffness distribution characteristics. A previous static biomechanical study indicated that the stronger the stabilizing effect of internal fixation, the lower the stress on endplates and cages (Xie et al., 2025). In this study, supplemental internal fixations were shown to effectively decrease both the dynamic response values and vibration amplitudes of the endplate and cage under WBV. Among the systems evaluated, BPS—regarded as the gold standard for OLIF supplementation—demonstrates well-documented biomechanical and clinical advantages (Wang et al., 2024; Dai et al., 2025; Xie et al., 2025). By utilizing a three-column fixation design, BPS effectively redirects WBV loads from the anterior and middle spinal columns (including the cage region) toward the posterior column through bilateral pedicle screws. This distributed load-transfer mechanism results in notable stress reduction within the surgical segment. In our study, the peak stress on the L5 superior endplate decreased by 59.58% in the BPS model relative to the SA model (from 4.2337 MPa to 1.7111 MPa), while the VA dropped from 0.7834 MPa to 0.3106 MPa. These observations are consistent with the finite element analysis by Xie et al., which indicated that BPS bears approximately 70% of the applied stress under both static and WBV conditions, further validating its efficient load-sharing capability (Xie et al., 2025). The OLLPS system, developed by our research team, creates a biomechanically optimized construct through locking integration of an anatomical plate with reverse pedicle screws. Although the stress-reducing capacity of OLLPS remained slightly inferior to that of BPS, it demonstrated markedly better dynamic stress regulation than the LP-2 and LP-4 models, with performance improvements of up to 73.68% and 57.08%, respectively. Analysis of implant stress responses revealed a consistent relationship that greater stabilization of the surgical segment corresponded to higher stress within the fixation device itself. The peak stress in OLLPS exceeded that of BPS, which may be attributed to the structural configuration of each system. While BPS distributes loads across two independent screw-rod constructs, OLLPS functions as an integrated screw-plate unit, resulting in more concentrated stress. Notably, despite the elevated stress levels in OLLPS with a Max of 52.2240 MPa, this represents only 10.44% of the fatigue strength of Ti-6Al-4V titanium alloy, indicating an adequate safety margin at the material level. Moreover, the locking mechanism of OLLPS directs loads efficiently into the lateral plate, mitigating stress concentration and promoting a more uniform stress distribution. This structural behavior further supports the mechanical safety and durability of the system (Wang et al., 2024). The screw tails of the LP-2 and LP-4 systems achieve fixation with the plate, relying solely on friction. Under the axial cyclic compressive loading induced by WBV, this structure tends to cause a “seesaw effect”, leading to excessive stress concentration at the screw-plate interface. This mechanical behavior was further verified by analyzing the dynamic peak stresses and stress nephograms. Thus, compared with the OLLPS and BPS systems, the LP-2 and LP-4 systems may be associated with a higher risk of failure under cyclic WBV loading.

### 4.3 Compensatory patterns and risks of adjacent segment dynamic responses

Lumbar fusion surgery disrupts the native mechanical equilibrium of the spine, leading to the gradual establishment of a new load-sharing pattern. Okuda et al. reported that decreased motion at the surgical site led to increased motion and load-bearing in adjacent segments (Okuda et al., 2021). Similarly, Pereira et al. demonstrated in the cadaveric models that adjacent segments experienced greater ROM and intervertebral disc strain (de Andrada Pereira et al., 2022). The core mechanisms of ASD after lumbar fusion surgery are motion compensation and load redistribution, and WBV amplifies this process through a resonance effect (Chen et al., 2001; Fan et al., 2018; Fan and Guo, 2021). Biomechanical alterations in adjacent discs can induce stress concentration, triggering a pathological cascade that includes tissue lesions, annular fissures, endplate calcification, and ultimately, cellular dysfunction and accelerated degeneration. A finite element analysis by Du et al. suggested that elevated stress in the adjacent disc following OLIF may be a key initiator of segmental degeneration (Du et al., 2021). Our study verified that all dynamic response indices of adjacent intervertebral discs in surgical models were higher than those in the intact model, and further found that peak stress concentrated at the posterior edge of the intervertebral disc. This mechanical characteristic aligns well with the clinical observation that the posterior edge of the intervertebral disc is more prone to annulus fibrosus injury and nucleus pulposus herniation, thereby providing more specific mechanical evidence for explaining the mechanism of adjacent segment degeneration after OLIF. Moreover, we observed a consistent biomechanical pattern across all surgical models: under identical loading conditions, the L3/4 disc exhibited consistently higher dynamic stress than the L5/S1 disc. These results suggest that, under cyclic WBV loading, the superior adjacent intervertebral segment carries a higher risk of degeneration than the inferior one after OLIF. This observation aligns with the static biomechanics research of Chen et al., who reported greater mechanical loading on the superior adjacent disc compared with the inferior one (Chen et al., 2001). Clinical support for this inference comes from Liu et al., who documented a greater propensity for early degenerative changes in superior adjacent segments following L4/5 OLIF (Liu et al., 2025). With the increasing stiffness of the four internal fixation systems (LP-2, LP-4, OLLPS, and BPS), the adjacent intervertebral disc stress showed a synchronous upward trend.​ The essence of the differential effects exerted by different internal fixation systems on adjacent segments lies in how their stiffness matches the spine’s biomechanical properties and the degree to which they restrict surgical segment movement. Further evidence comes from Chou’s study (Chou et al., 2025), where replacing the BPS titanium rod with a semirigid rod (polycarbonate urethane) alleviated both the abnormal increase in ROM and the rise in intervertebral disc stress of the upper adjacent segment, and remarkably reduced the biomechanical interference of the internal fixation on the adjacent segment. Notably, existing studies have confirmed that the use of an excessively rigid fixation method in spinal surgery not only accelerates the degeneration process of adjacent segments but also may cause the absorption of bone grafts in the vertebral bodies corresponding to the fixed segments, which in turn exerts an adverse impact on postoperative spinal stability and the outcome of bony fusion (Ha et al., 1993; Kang et al., 2023).

Altered stress patterns in adjacent facet joints may accelerate their degeneration, potentially increasing the risk of adjacent segment degeneration (Liu et al., 2025; Xie et al., 2025). Following OLIF surgery, the mechanical loading on these joints is substantially altered. In our study, the dynamic stress responses of adjacent facet joints mirrored the trends observed in adjacent intervertebral discs. Both the surgical intervention and supplemental internal fixation elevated facet joint stress, with the superior facets consistently sustaining higher stress than their inferior counterparts. Notably, when we analyzed the stress change rules of adjacent intervertebral discs and facet joints together, a clear pattern emerged: at the L3/4 segment, the four internal fixation systems (LP-2, LP-4, OLLPS, and BPS) caused stress to increase gradually, with slight differences between the systems; at the L5/S1 segment, however, stress increased in a stepwise manner, and the differences between the various internal fixation systems were much more obvious. Overall, BPS exerted the most negative biomechanical effects on both superior and inferior adjacent segments, with the impact being particularly marked at L5/S1. By comparison, OLLPS demonstrated a relative advantage in terms of stiffness buffering. The broader clinical implication of our findings is that for patients undergoing L4/5 OLIF with pre-existing degeneration at L5/S1, the choice of fixation method and extent of fusion requires careful consideration. When there is a pre-existing degenerative basis in the segment below the surgical level, the use of an internal fixation system with excessively strong biomechanical stability (such as BPS) may further exacerbate the stress abnormality in this segment, thereby potentially promoting its degenerative progression. In clinical practice, the selection of a supplemental fixation strategy should be guided by a comprehensive evaluation of the implant’s stiffness, its biomechanical compatibility with the native spinal segment, and the preoperative status of adjacent levels. Prioritizing absolute stability alone may not represent the optimal approach; rather, a strategy that balances adequate stabilization with favorable biomechanical compatibility appears more effective in mitigating the risk of postoperative adjacent segment complications.

This finite element study, with rigorous variable control and validation, yields robust findings despite certain limitations. First, the model assumed homogeneous, isotropic linear elastic materials—a standard simplification in comparative biomechanics. While viscoelasticity and degenerative features were not incorporated, this omission was necessary to isolate the core objective and does not compromise the validity of relative comparisons across fixation systems. Second, a uniform cage specification was used across models to ensure comparability; the effects of cage size, angle, or position were intentionally not explored, reflecting a focused scope rather than a flaw. Finally, although the loading conditions simulated clinically relevant daily vibration scenarios, they were restricted to short-duration exposure and failed to fully replicate the cumulative cyclic loading effects that persist over days, weeks, or even months in real postoperative environments. These limitations offer future research directions without diminishing the study’s scientific or clinical value.

## 5 Conclusion

This study concludes that different supplemental fixation strategies for OLIF induce distinct dynamic biomechanical responses at the surgical and adjacent segments. Compared with the LP-2, LP-4 and BPS systems, the OLLPS system demonstrates a more balanced biomechanical performance, providing adequate stability while causing less disturbance to adjacent segments. Clinically, OLIF fixation selection should account for preoperative adjacent segment conditions and avoid excessive rigidity to reduce the risk of postoperative adjacent segment degeneration.

## Data Availability

The original contributions presented in the study are included in the article/supplementary material, further inquiries can be directed to the corresponding author.
